# Alpelisib-Induced Diabetic Ketoacidosis in a Non-diabetic Patient

**DOI:** 10.7759/cureus.19295

**Published:** 2021-11-05

**Authors:** Tyler Fugere, Arya Mariam Roy, Issam Makhoul

**Affiliations:** 1 Internal Medicine, University of Arkansas for Medical Sciences, Little Rock, USA; 2 Hematology and Medical Oncology, Roswell Park Comprehensive Cancer Center, Buffalo, USA; 3 Hematology and Medical Oncology, University of Arkansas for Medical Sciences, Little Rock, USA

**Keywords:** pi3k inhibitors, primary breast malignancy, pik3ca gene, diabetic ketoacidosis (dka), alpelisib

## Abstract

Alpelisib is a phosphoinositol-3-kinase alpha catalytic subunit (PIK3CA) inhibitor used in patients with PIK3CA mutated breast cancer. The phosphatidylinositol 3-kinase (PI3K) pathway is responsible for activating protein kinase-B (AKT), and activated AKT promotes translation of glucose transporter 4 and glycogen synthesis in insulin-responsive tissues. Therefore, it is perhaps not surprising that hyperglycemia is the most common side effect of alpelisib, though diabetic ketoacidosis (DKA) appears to be a rare complication. This case describes the unique presentation of a patient with no prior history of diabetes who presented with DKA after starting alpelisib, and returned to euglycemia off of insulin just three days after stopping the drug suggesting that alpelisib can cause DKA in patients who did not previously have diabetes, and that the hyperglycemia is completely reversible upon discontinuation of the PIK3CA inhibitor and consequent restoration of the PI3K/AKT pathway.

## Introduction

Alpelisib is a phosphoinositol-3-kinase alpha catalytic subunit (PIK3CA) inhibitor recently approved by the FDA for use in patients with PIK3CA mutated breast cancer [1].The most common side effect of alpelisib is hyperglycemia, though diabetic ketoacidosis (DKA) appears to be rare. This case describes the unique presentation of a patient with no prior history of diabetes who presented with DKA after starting alpelisib, and returned to euglycemia off of insulin just three days after stopping alpelisib.

## Case presentation

A 48-year-old African American female presented to the emergency department with abdominal pain, nausea, and vomiting. Her medical history was significant for stage IIB estrogen receptor (ER) positive, progesterone receptor (PR) positive, human epidermal growth factor receptor two (HER2) negative left breast invasive ductal carcinoma diagnosed in 2011. She was treated with neoadjuvant chemotherapy, left mastectomy, radiation to the chest wall, and tamoxifen. Later, in 2018, she was found to have a recurrence of breast cancer with metastatic lesions to bone, abdomen, and lungs with a left-sided malignant pleural effusion. She received multiple lines of hormonal therapy and chemotherapy including goserelin, palbociclib, letrozole, fulvestrant, doxorubicin, and everolimus. Unfortunately, her disease continued to progress. In July 2020, Foundation One testing revealed PIK3CA mutation so alpelisib 300 mg daily was started. She continued to take letrozole, fulvestrant, and goserelin as well. She had no personal history of diabetes, and her hemoglobin A1c was 5.7% in July 2020 prior to initiating alpelisib. 

After 26 days of starting alpelisib, she developed abdominal pain, nausea, and vomiting and presented to the emergency department. Initial labs are displayed in Table 1. She was found to be in DKA with high anion gap metabolic acidosis. Infectious workup was negative and cardiac workup was not suggestive of any ischemic event. Chest x-ray revealed known left-sided pleural effusion. She was started on an insulin drip and admitted to the intensive care unit for management of DKA. Endocrinology was consulted for new-onset diabetes, which was attributed to the use of the PIK3CA inhibitor. Alpelisib was stopped, the anion gap and bicarbonate levels normalized with the administration of insulin drip and fluids, and she was successfully transitioned off the insulin drip to long-acting insulin nightly with sliding scale insulin at mealtime. However, she became hypoglycemic requiring discontinuation of all subcutaneous insulin, and no antihyperglycemic medications or subcutaneous insulin were continued at discharge from the hospital.

**Table 1:** Initial lab values

Component	Value (Reference)
WBC	10 K/uL (3.6- 9.50 K/uL)
Hemoglobin	10.9 g/dL (12.0-15 g/dL)
Platelet	415 K/uL (150-450 K/uL)
Sodium	136 mmol/L (135-145 mmol/L)
Potassium	4.0 mmol/L (3.5-5.1 mmol/L)
Chloride	107 mmol/L (98-107 mmol/L)
Bicarbonate	10 mmol/L (22-32 mmol/L)
Urea	4 mg/dL (6-20 mg/dL)
Creatinine	0.9 mg/dL (0.4-1.0 mg/dL)
Blood Glucose	302 mg/dL (70-110 mg/dL)
Total bilirubin	1.2 mg/dL (0.2-1.2 mg/dL)
Aspartate aminotransferase (AST)	40 IU/L (15-41 IU/L)
Alanine aminotransferase (ALT)	26 IU/L (4-45 IU/L)
Alkaline phosphatase	150 IU/L (32-91 IU/L)
Urine glucose	1000 mg/dL (negative)
Urine ketones	150 mg/dL (negative)
Beta-hydroxybutarate	7.58 mmol/L (<0.27 mmol/L)
Hemoglobin A1c	8.8% (4.0-6.0%)

## Discussion

Mutations in PIK3CA occur in around 30% of ER positive breast cancers and have a favorable prognostic effect in postmenopausal women with ER positive disease [2,3]. These gain of function mutations are associated with older age at diagnosis, hormone receptor positivity, HER2 negativity, lower tumor grade and stage, and lymph node negativity [3]. On May 24, 2019, the FDA approved alpelisib (PIQRAY®, Novartis Pharmaceuticals Corporation), a PIK3CA inhibitor, to be used in combination with fulvestrant for postmenopausal women and men with hormone receptor positive, HER2 negative, PIK3CA mutated advanced breast cancer [4]. FDA approval was based on the SOLAR-1 trial (NCT02437318), a phase three, randomized, double-blind, placebo-controlled trial of alpelisib plus fulvestrant versus placebo plus fulvestrant [4]. The results of the trial were significant for progression-free survival of 11 months in the alpelisib-fulvestrant group compared to 5.7 months in the placebo-fulvestrant group. 

The phosphatidylinositol 3-kinase (PI3K) pathway is responsible for activating protein kinase-B (AKT), and activated AKT promotes translation of glucose transporter 4 and glycogen synthesis in insulin-responsive tissues [5]. In the SOLAR-1 study, the most frequent adverse event of any grade was hyperglycemia, which occurred in 63.7% of the alpelisib-fulvestrant group versus 9.8% of the placebo-fulvestrant group. Hyperglycemia was also the most common grade three or four adverse event, occurring in 36.6% of the patients who received alpelisib-fulvestrant and 0.7% of those who received placebo-fulvestrant [4]. Hyperglycemia was the most common adverse event in other alpelisib studies as well. In a phase 1b clinical trial published in 2019 comparing alpelisib plus fulvestrant in PIK3CA-altered and PIK3CA-wild-type ER positive advanced breast cancer, the maximum tolerated dose was 400 mg of alpelisib, and the most frequent adverse event was hyperglycemia, which occurred in 22% of patients [6]. 

According to the “Keeping TRAQ with PIQRAY: Patient Management Tool” on Novartis’s website, ketoacidosis was reported in 0.7% of patients (n=2) who received alpelisib [7]. Patients with type one diabetes and uncontrolled type two diabetes were excluded from the SOLAR-1 trial, but patients with a history of type two diabetes were included. It is not reported if the two patients in the study who developed ketoacidosis had a medical history of type two diabetes. 

The patient discussed in our case had a hemoglobin a1c level of 5.7% prior to initiation of alpelisib, checked on the same day the medication was prescribed, yet she presented to the emergency department in DKA 26 days after starting the medication. The American Diabetes Association (ADA) recommends starting insulin-naive patients on 0.5-0.8 units per kilogram of body weight per day divided into regular or rapid-acting and basal insulin after resolution of DKA [8]. The patient in this case weighed 50 kg, but was transitioned off of the insulin drip to just five units of glargine daily, which ultimately had to be discontinued altogether as she became hypoglycemic. Alpelisib was not restarted, all insulin therapy was stopped, and she did not have any more episodes of hyperglycemia. 

## Conclusions

This is a case of a patient with no known personal history of diabetes who presented to the hospital in DKA after starting the PIK3CA inhibitor alpelisib for treatment of metastatic breast cancer. Alpelisib is known to cause hyperglycemia by inhibiting the PI3K/AKT pathway, which is responsible for the translation of glucose transporter 4 and for glycogen synthesis in insulin-responsive tissues. Once alpelisib was stopped, our patient returned to euglycemia and did not require any long-term supplemental insulin despite presenting in DKA. This case demonstrates that inhibition of the PI3K/AKT pathway by alpelisib can be so dramatic that it can cause DKA in patients who did not previously have diabetes and that the hyperglycemia is completely reversible upon discontinuation of the PIK3CA inhibitor.

## References

[1] (2021). U.S. Food and Drug Administration: FDA approves alpelisib for metastatic breast cancer. https://www.fda.gov/drugs/resources-information-approved-drugs/fda-approves-alpelisib-metastatic-breast-cancer.

[2] Ellis MJ, Lin L, Crowder R (2010). Phosphatidyl-inositol-3-kinase alpha catalytic subunit mutation and response to neoadjuvant endocrine therapy for estrogen receptor positive breast cancer. Breast Cancer Res Treat.

[3] Kalinsky K, Jacks LM, Heguy A (2009). PIK3CA mutation associates with improved outcome in breast cancer. Clin Cancer Res.

[4] André F, Ciruelos E, Rubovszky G (2019). Alpelisib for PIK3CA-mutated, hormone receptor-positive advanced breast cancer. N Engl J Med.

[5] Huang X, Liu G, Guo J, Su Z (2018). The PI3K/AKT pathway in obesity and type 2 diabetes. Int J Biol Sci.

[6] Juric D, Janku F, Rodón J (2019). Alpelisib plus fulvestrant in PIK3CA-altered and PIK3CA-wild-type estrogen receptor-positive advanced breast cancer: a phase 1b clinical trial. JAMA Oncol.

[7] (2021). Keeping TRAQ with PIQRAY: Patient Management Tool. https://www.hcp.novartis.com/siteassets/piqray/piqray-advanced-breast-cancer-interactive-patient-management-tool.pdf.

[8] Kitabchi AE, Umpierrez GE, Murphy MB, Kreisberg RA (2006). Hyperglycemic crises in adult patients with diabetes: a consensus statement from the American Diabetes Association. Diabetes Care.

